# Feasibility of liquid biopsy using plasma and platelets for detection of anaplastic lymphoma kinase rearrangements in non-small cell lung cancer

**DOI:** 10.1007/s00432-019-02944-w

**Published:** 2019-06-01

**Authors:** Cheol-Kyu Park, Ji-Eun Kim, Min-Seok Kim, Bo-Gun Kho, Ha-Young Park, Tae-Ok Kim, Hong-Joon Shin, Hyun-Joo Cho, Yoo-Duk Choi, In-Jae Oh, Young-Chul Kim

**Affiliations:** 1 Department of Internal Medicine, Chonnam National University Medical School, Gwangju, Republic of Korea; 2 Lung and Esophageal Cancer Clinic, Chonnam National University Hwasun Hospital, 322 Seoyang-ro, Hwasun, Jeonnam 58128 Republic of Korea; 3 Department of Pathology, Chonnam National University Medical School, Gwangju, Republic of Korea

**Keywords:** Liquid biopsy, Plasma, Platelets, Anaplastic lymphoma kinase, Non-small cell lung cancer

## Abstract

**Purpose:**

Fluorescence in situ hybridization (FISH) using tumor tissue is the gold standard for detection of anaplastic lymphoma kinase (*ALK*) rearrangement in non-small cell lung cancer (NSCLC). However, this method often is not repeatable due to difficulties in the acquisition of tumor tissues. Blood-based liquid biopsy using reverse transcription polymerase chain reaction (RT-PCR) is expected to be useful to overcome this limitation. Here, we investigated the feasibility of liquid biopsy using plasma and platelets for detection of *ALK* rearrangement and prediction of ALK inhibitor treatment outcomes.

**Methods:**

*ALK*-FISH assays were performed in 1128 tumor specimens of NSCLC between January 2015 and June 2018. We retrospectively analyzed formalin-fixed paraffin-embedded (FFPE) tissues from previously confirmed FISH-positive (*n* = 199) and -negative (*n* = 920) cases. We recruited patients who had available tissue specimens and agreed to venous sampling. RNA was extracted from FFPE blocks, plasma, and platelets. Fusion RNA of echinoderm microtubule-associated protein-like 4 (*EML4*)-*ALK* was detected by quantitative PCR.

**Results:**

Thirty-three FISH-positive and 28 FISH-negative patients were enrolled. In validation, data compared with FISH, RT-PCR using FFPE tissues showed 54.5% sensitivity, 78.6% specificity, and 75.5% accuracy. Liquid biopsy had higher sensitivity (78.8%), specificity (89.3%) and accuracy (83.6%). Higher positivity for liquid biopsy was shown in subgroups with delayed (≥ 6 months from diagnosis) blood sampling (plasma, 85.7%; platelets, 87.0%). In 26 patients treated with crizotinib, the platelet-positive subgroup showed longer median duration of treatment (7.2 versus 1.5 months), longer median progression-free survival (5.7 months versus 1.7 months), a higher overall response rate (70.6% versus 11.1%), and a higher disease control rate (88.2% versus 44.4%) than the platelet-negative subgroup.

**Conclusion:**

Liquid biopsy could have applications in the diagnosis of ALK-positive NSCLC, even when using RT-PCR, and platelets can be useful for predicting treatment outcomes of ALK inhibitors.

## Introduction

Rearrangements in the anaplastic lymphoma kinase (*ALK*) gene occur in 3–7% of patients with non-small cell lung cancer (NSCLC) (Shaw et al. 2009), and ALK-positive lung cancer has been defined as a distinct clinical and molecular subtype of NSCLC (Lin et al. 2017; Shaw et al. 2009; Soda et al. 2007). NSCLCs harboring *ALK* rearrangements are ALK-dependent for growth and survival, and show marked sensitivity to treatment with ALK tyrosine kinase inhibitors (TKIs), such as crizotinib (Shaw et al. 2013; Solomon et al. 2014), ceritinib (Shaw et al. 2017; Soria et al. 2017), and alectinib (Novello et al. 2018; Peters et al. 2017). Thus, according to recent clinical trials, ALK-positive lung cancer could be considered as the best subgroup in advanced-stage NSCLCs, showing good long-term survival when ALK TKIs are given as first-line treatment and continue to subsequent treatment (Peters et al. 2017; Solomon et al. 2018). Therefore, routine testing for *ALK* gene rearrangement is recommend in all patients with non-squamous NSCLC (Hanna et al. 2017; Non-small cell lung cancer (Version 6.2018) 2018; Planchard et al. 2018).

Fluorescence in situ hybridization (FISH) using tissue biopsy specimens is the gold standard for the detection and confirmation of *ALK* rearrangement by break-apart assay, which yields signals for the fusion *ALK* gene. Immunohistochemistry (IHC) is widely used and also FDA-approved diagnostic test to identify ALK protein, and correlation between positive ALK IHC and a positive *ALK*-FISH is over 90% in general (Kerr and Lopez-Rios 2016; Lindeman et al. 2018). However, this method often is not repeatable due to difficulties in the acquisition of tumor tissues (Kerr and Lopez-Rios 2016). In addition, rebiopsy and analysis of acquired mutations within the ALK tyrosine kinase domain have been highlighted in patients who relapsed after first-line ALK TKI treatment in the era of next-generation ALK TKIs (Dagogo-Jack et al. 2018; Gainor et al. 2016; Lin et al. 2017). Blood-based liquid biopsy using reverse-transcription polymerase chain reaction (RT-PCR) is expected to overcome these limitations and may permit frequent assessment with monitoring of biomarkers (Nilsson et al. 2016; Perez-Callejo et al. 2016). Several reports have described liquid biopsy for the detection of *ALK* rearrangement (Ilie et al. 2012; Li et al. 2017; Nilsson et al. 2016; Pailler et al. 2013; Rolfo et al. 2017). Among the sources of blood-based liquid biopsy, platelets have been shown to provide valuable information regarding the tumor by sequestering RNA released as circulating microvesicles from the tumor. Moreover, platelets can be immediately isolated and can undergo repetitive examinations for serial monitoring of biomarkers using RT-PCR (Best et al. 2015; Nilsson et al. 2011; Nilsson et al. 2016).

However, although liquid biopsy has many advantages compared with tissue biopsy, there are several limitations with regard to the application of blood-based liquid biopsy in ALK-positive NSCLC. In fact, detection techniques have not been standardized according to the sources of liquid biopsy, and commercial kits for routine use of liquid specimens, particularly by RT-PCR, are limited, unlike the situation in the detection of epidermal growth factor receptor (*EGFR*) activating and resistance mutations. Recently, next-generation sequencing (NGS) using a commercial platform for liquid biopsy has expanded the scope of applications for the detection of acquired mutations and for diagnosis in ALK-positive NSCLC (Beadling et al. 2016; Cui et al. 2017; Gainor et al. 2016; Lin et al. 2017; Nilsson et al. 2016; Rolfo et al. 2017; Wang et al. 2016; Yoda and Lin 2018). However, the wide application of liquid NGS is limited owing to the need for specialized equipment and the high costs of the method.

In this study, we aimed to assess the feasibility of blood-based liquid biopsy using plasma and platelets for detection of *ALK* rearrangement by RT-PCR with commercial kits initially developed for tissue genotyping. In addition, we investigated the clinical characteristics of patients according to the positivity of liquid biopsy and the predictive value of blood-based liquid biopsy for ALK inhibitor treatment.

## Methods and materials

### Patients and sample collection

**Fig. 1:**
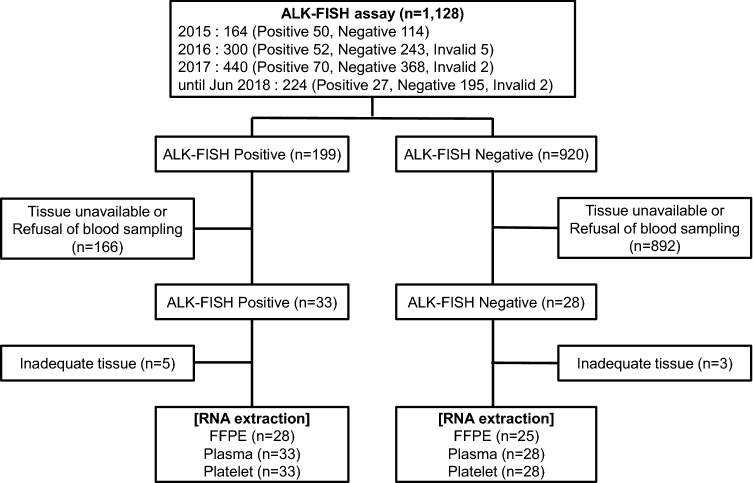
Patient enrollments. *FISH* fluorescence in situ hybridization; *FFPE* formalin-fixed paraffin-embedded

FISH assays for the detection of *ALK* rearrangement were performed using tumor specimens from 1,128 patients with NSCLC between January 2015 and June 2018. We retrospectively analyzed formalin-fixed paraffin-embedded (FFPE) tissues from previously confirmed FISH-positive (*n* = 199) and -negative (*n* = 920) cases. We recruited patients who had available tissue specimens and agreed to venous sampling (Fig. 1). Tumor samples were obtained from FFPE sections from tumor tissues and FFPE cell blocks from cytology specimens obtained from bronchoscopic procedures (washing or brushing) or malignant pleural effusions. Tumor samples for RT-PCR were collected from the same specimens used for FISH assays. Blood samples were obtained at diagnosis, before or during systemic treatment with cytotoxic chemotherapy and ALK TKIs. For analysis of serial monitoring during ALK TKI treatment, enrolled patients administered ALK TKIs were required to provide a blood sample at every visit, and venous sampling was performed on weeks 1, 4, and 8 and then every 2–3 months in parallel with imaging studies for response assessment.

### Detection of *ALK* gene rearrangement by FISH assays in tumor tissues

**Fig. 2:**
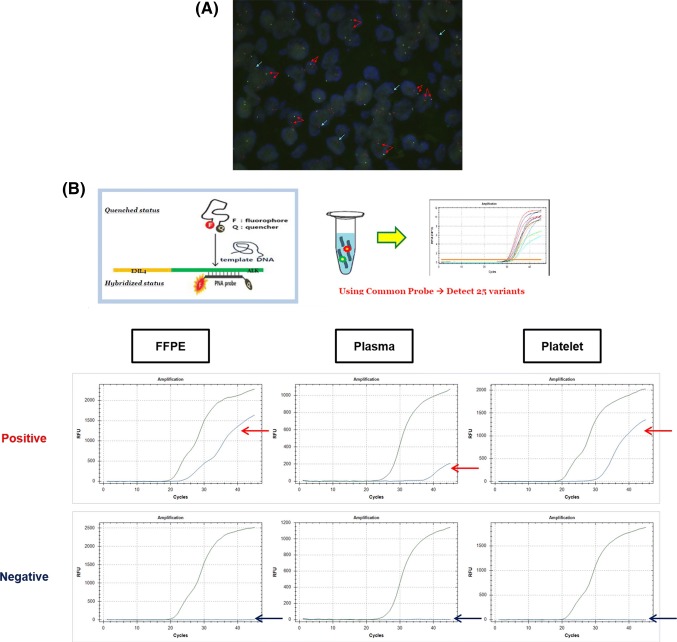
Representative images of *ALK* rearrangements. **a** FFPE tissue FISH assay. FISH-positive cells were defined as showing split signals (red arrows) or an isolated red signal (blue arrows). FISH-positive cases were defined as cases with more than 15% tumor cells showing signals. **b** RT-PCR: FFPE tissue, plasma, platelets. **a**, **b** These images are from the case in Fig. 4a. *FFPE* formalin-fixed paraffin-embedded, *FISH* fluorescence in situ hybridization, *RT*-*PCR* reverse transcription polymerase chain reaction

*ALK* rearrangement was detected by FISH assays using a break-apart probe specific for the ALK locus (Vysis LSI ALK dual-color, break-apart rearrangement probe; Abbott Molecular, Abbott Park, IL, USA) in FFPE tumor tissue samples. FISH-positive samples were defined as those with more than 15% of tumor cells showing split signals or an isolated red signal (3′ signal) as described in the previous studies (Fig. 2a) (Paik et al. 2011; Shaw et al. 2009).

### Isolation of plasma and platelets

Plasma and platelets were isolated from the same sample of whole blood in 10-mL purple-cap BD Vacutainers containing ethylenediaminetetraacetic acid (EDTA) anticoagulant, and Eppendorf centrifugation (5810R) was carried out immediately before storage within 2 h from venous sampling. The cells and aggregates were removed by centrifugation at 4 °C for 10 min at 700×*g*, yielding platelet-rich plasma. The platelets were isolated from the platelet-rich plasma by centrifugation at 4 °C for 10 min at 1700×*g*, and the plasma was then collected as an aliquot from the supernatant and frozen at − 80 °C for further use. The platelet pellet was collected and mixed with 300 μL RNAlater solution (Life Technologies, Carlsbad, CA, USA) and frozen at − 80 °C for further use. The plasma and platelets were frozen in parallel.

### RNA extraction and cDNA synthesis

In case of FFPE tissues, RNA was extracted from 3 slides of 4–5–μm thickness taken from the FFPE blocks using the PureLink™ FFPE Total RNA Isolation Kit (Invitrogen, Carlsbad, CA, USA). RNA was also extracted from the plasma and platelets using a RiboPure-Blood Kit (Life Technologies) according to the manufacturers’ instructions. The resulting RNA was eluted in 50-μL elution buffer. The concentration and purity of the extracted RNA were determined using a NanoDrop ND-2000 spectrophotometer (Thermo Fisher Scientific, DE, USA). The quantity of total extracted RNA was only measured in FISH-positive patients. The extracted RNA was stocked at − 80 °C until use. We used 250-ng total RNA for cDNA synthesis using a SuperScript VILO cDNA Synthesis Kit (Life Technologies).

### Peptide nucleic acid (PNA)-mediated RT-PCR assay for *EML4*-*ALK* screening

The most common *ALK* rearrangement is characterized by fusion of the *ALK* gene with echinoderm microtubule-associated protein-like 4 (*EML4*); the fusion gene has multiple chimeric variants with the same portion of *ALK* and different truncations of *EML4* (Sasaki et al. 2010; Soda et al. 2007). The *EML4*-*ALK* fusion RNA was detected by PNA-mediated RT-PCR assays using a PANA EML4-ALK Fusion Gene Detection Screening Kit (Panagene, Daejeon, South Korea), which was initially developed for tissue genotyping. This assay was previously performed in a retrospective study for genotyping of *ALK* fusion variants and showed favorable performance in terms of sensitivity (57/81, 70.4%) and meaningful correlations with clinical implications, demonstrating that specific variants of *EML4*-*ALK* showed poor prognosis and multidrug resistance to ALK TKI treatment (Woo et al. 2017). This kit was designed to detect 28 types of known *ALK* rearrangements for screening, including E6;A19, E6;A20 (variant 3a), E6ins33;A20 (variant 3b, three subtypes), E6;ins18A20, E13;A20 (variant 1, five subtypes), E13;ins69A20 (variant 6, two subtypes), E20;A20 (variant 2, two subtypes), E20; ins18A20 (two subtypes), E14ins11; del49A20 (variant 4), E14; del12A20 (variant 7), E14; del36A20, E14ins2; ins56A20, E2; A20 (variant 5a), E2; ins117A20 (variant 5b), E17; ins30A20 (variant 8a), E17ins61; ins34A20 (variant 8b), E17ins65; A20, E17; ins68A20, and E17del58; ins39A20. PCR was performed under the following conditions: 2 min at 50 °C; 15 min at 95 °C; five cycles of 10 s at 95 °C and 30 s at 58 °C; and 45 cycles of 10 s at 95 °C, 30 s at 58 °C, and 15 s at 72 °C. The assay results were interpreted as positive for *EML4*-*ALK* according to the manufacturer’s instructions. A positive result was defined as a threshold cycle (Ct) value of less than 40, and the internal control was defined as a Ct value less than 36. The assay results were regarded as invalid if the assays for the *EML4*-*ALK* fusion gene and internal control all showed simultaneously negative results. When an invalid result for RT-PCR was obtained, the assay was repeated using newly synthesized cDNA (Fig. 2b).

### Statistical analysis

Treatment responses for chemotherapy and ALK TKIs were evaluated according to the revised RECIST version 1.1 (Eisenhauer et al. 2009). Progression-free survival (PFS) was defined as the time (in months) from the first date of chemotherapy or ALK TKI treatment until the date of objective progression of disease or death from any cause.

We collected clinical information of enrolled patients at diagnosis and during the chemotherapy or ALK TKI treatment. All data are expressed as means ± standard deviations (SD), median (range), or numbers with percentages. Intergroup comparisons were performed using the Mann–Whitney *U* test for continuous variables and Pearson’s *χ*^2^ test or Fisher’s exact test for categorical variables. Survival times were estimated for each group using the Kaplan–Meier method. Statistical analysis was performed with IBM SPSS statistics version 25 (SPSS, Inc., an IBM Company, Chicago, IL, USA), and differences with *p* values of less than 0.05 were considered as statistically significant.

## Results

### Baseline characteristics

Excluding patients who did not have available tissue specimens or refused to provide blood samples, 33 patients with FISH-positive results and 28 patients with FISH-negative results were finally enrolled. RNA extraction was performed in each group according to the experimental protocol, and cases with inadequate tissue for analysis were excluded (Fig. 1). The mean age of enrolled patients was 63.7 (± 10.7), and the FISH-positive subgroup had younger patients than the FISH-negative group. Moreover, the FISH-positive subgroup had more patients with brain metastasis and brain radiation therapy. There were no differences in sex, smoking history, *EGFR* mutation status, or response to pemetrexed treatment between the two subgroups (Table 1).

**Table 1:** Comparison of baseline characteristics of patients according to FISH results

Characteristics, *n* (%)	Total (*n* = 61)	FISH-positive (*n* = 33)	FISH-negative (*n* = 28)	*p*
Age, years, mean (SD)	63.7 (10.7)	61.3 (10.9)	66.5 (9.8)	0.076
Sex				0.178
Male	34 (55.7)	21 (63.6)	13 (46.4)	
Female	27 (44.3)	12 (36.4)	15 (53.6)	
Smoking				0.873
Ever-smoker	29 (47.5)	16 (48.5)	13 (46.4)	
Never-smoker	32 (52.5)	17 (51.5)	15 (53.6)	
Histology				0.416
ADC	59 (96.7)	31 (93.9)	28 (100.0)	
SQC	1 (1.6)	1 (3.0)	0 (0.0)	
ADSQC	1 (1.6)	1 (3.0)	0 (0.0)	
Differentiation				0.358
Well	9 (14.8)	3 (9.1)	6 (21.4)	
Moderate	13 (21.3)	9 (27.3)	4 (14.3)	
Poor	26 (42.6)	15 (45.5)	11 (39.3)	
Not evaluable	13 (21.3)	6 (18.2)	7 (25.0)	
EGFR mutation-positive	7 (11.4)	4 (12.1)	3 (10.7)	0.221
Stage at diagnosis				0.752
IIIA/IIIB	3 (4.9)/2 (3.3)	1 (3.0)/1 (3.0)	2 (7.1)/1 (3.6)	
IV	56 (91.8)	31 (93.9)	25 (89.3)	
Intrathoracic	23 (41.1)	11 (35.5)	12 (48.0)	0.605
Extrathoracic (single)	13 (23.2)	9 (29.0)	4 (16.0)	0.217
Extrathoracic (multiple)	20 (35.7)	11 (35.5)	9 (36.0)	0.921
Brain metastasis	19 (31.1)	13 (39.4)	6 (21.4)	0.131
Brain RT	12 (19.7)	10 (30.3)	2 (7.1)	0.023
Pemetrexed-based chemotherapy	36 (59.0)	22 (66.7)	14 (50.0)	0.187
Line, median (range)	1 (1–3)	1 (1–3)	1 (1–1)	0.267
Duration, months, median (range)	3.5 (0.0–42.7)	3.9 (0.0–42.7)	3.5 (0.0–13.4)	0.141
PFS, months, median (95% CI)	3.9 (3.1–4.8)	3.9 (3.3–4.6)	1.5 (0.0–5.5)	0.182
Overall response rate	9/36 (25.0)	4 (18.2)	5 (35.7)	0.267
Disease control rate	26/36 (72.2)	18 (81.8)	8 (57.1)	0.140

*SD* standard deviation, *FISH* fluorescence in situ hybridization, *ADC* adenocarcinoma, *SQC* squamous carcinoma, *ADSQC* adenosquamous carcinoma, *EGFR* epidermal growth factor receptor, *RT* radiation therapy, *PFS* progression-free survival

### Detection of *ALK* rearrangements using RT-PCR

**Fig. 3:**
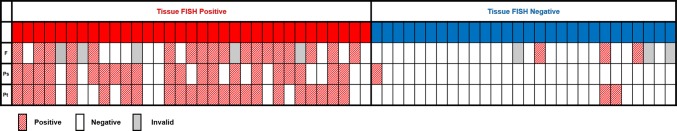
Experimental data about RT-PCR positivity according to biopsy resources. *F* accounts for FFPE tissue, *Ps* for plasma and *Pt* for platelets. *FFPE* formalin-fixed paraffin-embedded, *RT*-*PCR* reverse transcription polymerase chain reaction

The validation data compared with FISH for tumor tissues are described in Table 2, and representative images for FISH and RT-PCR are shown in Fig. 2. All experimental data about RT-PCR positivity according to biopsy resources are displayed in Fig. 3. RT-PCR using FFPE tissues showed 54.5% sensitivity, 78.6% specificity, and 75.5% accuracy. Liquid biopsy (plasma or platelets) had higher sensitivity (78.8%), specificity (89.3%) and accuracy (83.6%). Platelets showed slightly higher sensitivity than plasma (Table 2).

**Table 2:** Detection of ALK rearrangement using RT-PCR in tissue and liquid biopsy

RT-PCR, *n* (%)	FISH-positive (*n* = 33)	FISH-negative (*n* = 28)
FFPE		
Positive	18 (54.5)	3 (10.7)
Negative	10 (30.3)	22 (78.6)
Inadequate	5 (15.2)	3 (10.7)
Accuracy	40/53 (75.5)	
Plasma		
Positive	21 (63.6)	1 (3.6)
Negative	12 (36.4)	27 (96.4)
Accuracy	48/61 (78.7)	
Platelet		
Positive	23 (69.7)	2 (7.1)
Negative	10 (30.3)	26 (92.9)
Accuracy	49/61 (80.3)	
Liquid biopsy		
Positive^a^	26 (78.8)	3 (10.7)
Negative^b^	7 (21.2)	25 (89.3)
Accuracy	51/61 (83.6)	

*RT*-*PCR* reverse transcription polymerase chain reaction, *FISH* fluorescence in situ hybridization, *FFPE* formalin-fixed paraffin-embedded

^a^Plasma or platelets, ^b^Plasma and platelets

### Characteristics of ALK-positive NSCLC according to liquid biopsy positivity

A comparison of molecular and clinical characteristics in FISH-positive patients is shown in Table 3. Median proportions of positive cells in FISH were likely to be higher in subgroups of liquid biopsy-positive results (plasma, 20.0% versus 15.0%, *p* = 0.082; platelets 20.0% versus 15.0%, *p* = 0.084). All blood samples of liquid biopsy-positive subgroups were collected after initiation of systemic treatment, regardless of the types (chemotherapy or ALK TKI). However, platelet showed more liquid biopsy-negative result when the patients were treated with ALK TKI rather than chemotherapy (85.7% versus 14.3%, *p* = 0.077). Higher positivity for liquid biopsy was shown in the subgroup of delayed blood sampling since diagnosis more than 6 months (plasma, 85.7%, *p* = 0.106; platelets, 87.0%, *p* = 0.036). In a subgroup who had a blood sampling within 6 months, 62.5% of samples were drawn after start of systemic treatment (Table 4). In another subgroup who had a blood sampling after 6 months, all samples were drawn after start of systemic treatment. Positive rate of liquid biopsy was relatively higher in patients who were progressed after chemotherapy and preparing ALK TKI treatment than in those who were in ALK TKI treatment.

**Table 3:** Comparison of molecular and clinical characteristics in FISH-positive patients according to detection of *ALK* rearrangement using plasma or platelets

Characteristics, *n* (%)	Total (*N* = 33)	Plasma			Platelet		
		+ (*N* = 21)	− (*N* = 12)	*p*	+ (*N* = 23)	− (*N* = 10)	***p***
FISH-positive proportion, %, median (range)	15.0 (15.0–80.0)	20.0 (15.0–70.0)	15.0 (15.0–80.0)	0.082	20.0 (15.0–80.0)	15.0 (15.0–35.0)	0.084
Total RNA, ng/μL, mean (SD)							
FFPE/plasma/platelets		93.45 (124.10)/2.26 (0.63)/2.71 (1.08)					
Liquid		2.34 (0.61)	2.04 (0.66)	0.252	2.74 (1.21)	2.55 (1.10)	0.784
Time point at sampling							
Before initiation of systemic treatment	3 (9.1)	0 (0.0)	3 (25.0)	0.040	0 (0.0)	3 (30.0)	0.022
After initiation of systemic treatment	30 (90.9)	21 (100.0)	9 (75.0)	–	23 (100.0)	7 (70.0)	–
After CTx and before ALK TKI	13 (43.3)	9 (42.9)	4 (44.4)	0.936	12 (52.2)	1 (14.3)	0.077
After ALK TKI	17 (56.7)	12 (57.1)	5 (55.6)	–	11 (47.8)	6 (85.7)	–
Interval from diagnosis to sampling, months, median (range)	11.7 (5.9–21.7)	11.7 (3.7–120.0)	10.8 (0.0–73.5)	0.681	11.7 (1.6–120.0)	9.8 (0.0–57.8)	0.411
< 6 months, n (%)	8 (24.2)	3 (14.3)	5 (41.7)	0.106	3 (13.0)	5 (50.0)	0.036
≥ 6 months, n (%)	25 (75.8)	18 (85.7)	7 (58.3)	–	20 (87.0)	5 (50.0)	–
Treatment before crizotinib	30 (90.9)	21 (100.0)	9 (75.0)	0.040	23 (100.0)	7 (70.0)	0.022
Operation	6 (18.2)	4 (19.0)	2 (16.7)	1.000	5 (21.7)	1 (10.0)	0.640
RT	4 (12.1)	1 (4.8)	3 (25.0)	0.125	3 (13.0)	1 (10.0)	1.000
Chemotherapy	24 (72.7)	19 (90.5)	5 (41.7)	0.005	18 (78.3)	6 (60.0)	0.400
EGFR TKI	5 (15.2)	4 (19.0)	1 (8.3)	0.630	5 (21.7)	0 (0.0)	0.291
Prior Tx except operation	29 (87.9)	21 (100.0)	8 (66.7)	0.012	23 (100.0)	6 (60.0)	0.005
Treatment after crizotinib	16 (48.5)	10 (47.6)	6 (50.0)	0.476	11 (47.8)	5 (50.0)	0.107
Chemotherapy	8 (24.2)	5 (23.8)	3 (25.0)		4 (17.4)	4 (40.0)	
ALK TKI	11 (33.3)	8 (38.1)	3 (25.0)		9 (39.1)	2 (20.0)	
EGFR TKI	2 (6.1)	2 (9.5)	0 (0.0)		2 (8.7)	0 (0.0)	
ICI	3 (9.1)	3 (14.3)	0 (0.0)		2 (8.7)	1 (10.0)	
BSC	1 (3.0)	0 (0.0)	1 (8.3)		0 (0.0)	1 (10.0)	
ALK TKI—crizotinib	26 (78.8)	16 (76.2)	10 (83.3)	1.000	17 (73.9)	9 (90.0)	0.397
Line, median (range)	2 (1–6)	2 (1–6)	2 (1–3)	0.066	2 (1–6)	2 (1–3)	0.924
Duration, months, median (range)	5.6 (0.3–39.6)	5.3 (0.3–39.6)	4.0 (0.6–39.5)	1.000	7.2 (0.7–39.6)	1.5 (0.3–39.5)	0.090
PFS, months, median (95% CI)	5.2 (2.5–8.0)	5.4 (2.8–8.0)	4.0 (0.0–10.0)	0.713	5.7 (0.0–16.7)	1.7 (0.5–3.0)	0.028
Best response							
PR	13 (50.0)	9 (56.3)	4 (40.0)		12 (70.6)	1 (11.1)	
SD	6 (23.1)	3 (18.8)	3 (30.0)		3 (17.6)	3 (33.3)	
PD	6 (23.1)	4 (25.0)	2 (20.0)		2 (8.7)	4 (44.4)	
NE	1 (3.8)	0 (0.0)	1 (10.0)		0 (0.0)	1 (11.1)	
Overall response rate	13/26 (50.0)	9/16 (56.3)	4/10 (40.0)	0.420	12/17 (70.6)	1/9 (11.1)	0.011
Disease control rate	19/26 (73.1)	12/16 (75.0)	7/10 (70.0)	1.000	15/17 (88.2)	4/9 (44.4)	0.028

**Table 4:** Correlation between time point at blood sampling and interval from diagnosis to blood sampling

*n* (%)	Interval from diagnosis to sampling	
	< 6 months (*n* = 8)	≥ 6 months (*n* = 25)
Time point at sampling		
Before initiation of systemic treatment	3 (37.5)	0 (0.0)
Positive for plasma/platelet	0 (0.0)/0 (0.0)	0(0.0)/0 (0.0)
After initiation of systemic treatment	5 (62.5)	25 (100.0)
(1) After CTx and before ALK TKI	3 (37.5)	10 (40.0)
Positive for plasma/platelet	2 (66.7)/3 (100.0)	7 (70.0)/9 (90.0)
(2) After ALK TKI	2 (25.0)	15 (60.0)
Positive for plasma/platelet	1 (50.0)/0 (0.0)	11 (73.3)/11 (73.3)

*CTx* chemotherapy, *TKI* tyrosine kinase inhibitor

Among 26 patients treated with crizotinib, the platelet-positive subgroup showed longer duration of treatment (median, 7.2 versus 1.5 months, *p* = 0.090), longer median PFS [5.7 months, 95% confidence interval (CI) 0.0–16.7 versus 1.7 months, 95% CI 0.5–3.0; *p* = 0.028], a higher overall response rate (70.6% versus 11.1%, *p* = 0.011), and a higher disease control rate (88.2% versus 44.4%, *p* = 0.028) than the platelet-negative subgroup.

### Serial monitoring of *ALK* rearrangement during ALK TKI treatment

**Fig. 4:**
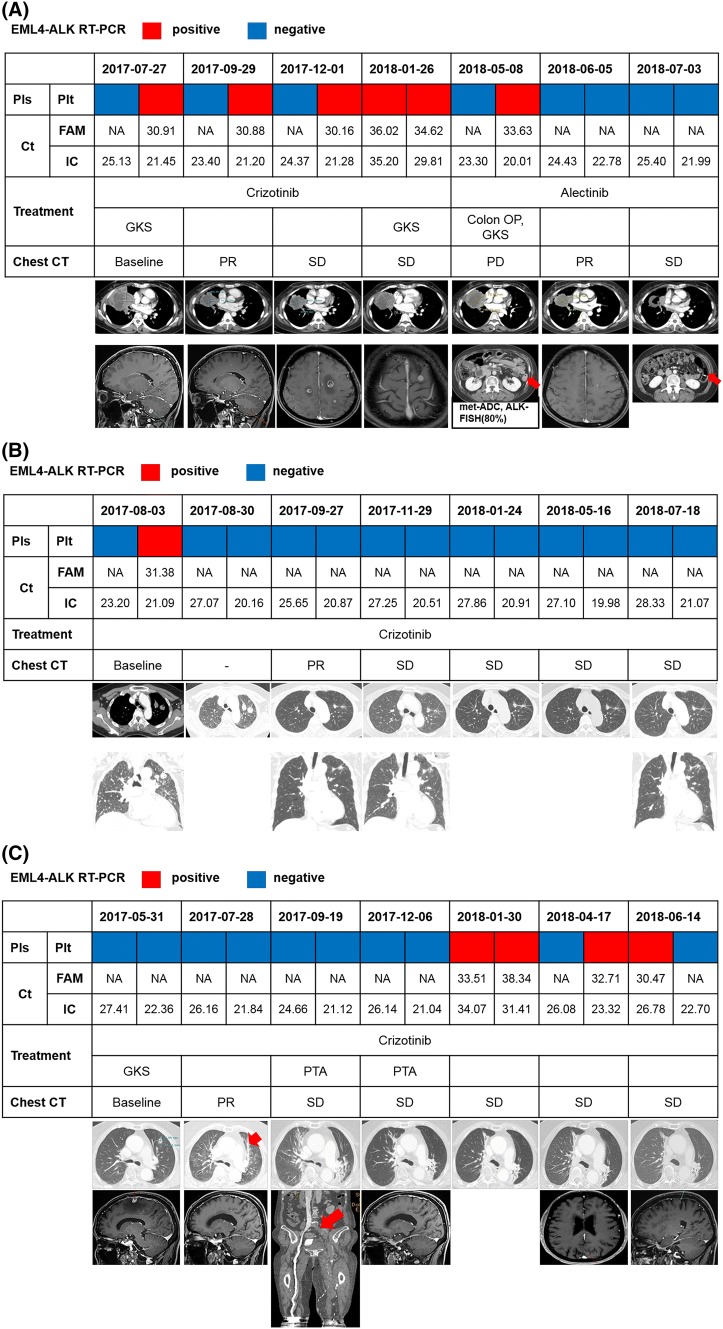
Serial monitoring using liquid biopsy for detection of ALK rearrangements. **a** Case #1. Brain metastasis had recurred during crizotinib treatment, along with continuous detection of ALK rearrangement in plasma and platelets. After debulking surgery for colon metastasis and initiation of alectinib treatment, negative conversion was shown in liquid biopsy. **b** Case #2. Loss of ALK rearrangement was sustained during crizotinib treatment. Tumor burden had decreased, consistent with these results of liquid biopsy. **c** Case #3. Initial brain imaging before CCRT did not show metastasis. *ALK* rearrangement was not detected in the blood, even after the first recurrence of the brain tumor. She received PTA due to deep vein thrombosis, and liquid biopsy showed positive conversion 3 months before the second recurrence of the brain tumor. Until then, she did not have any neurological symptoms or signs and maintained crizotinib treatment beyond progression. *RT*-*PCR* reverse transcription polymerase chain reaction, *Pls* plasma, *Plt*, platelet, *NA* not available, *FAM* EML4-ALK fusion typers, *IC* internal control, *GKS* gamma-knife surgery for brain, *OP* operation, *PR* partial remission, *SD* stable disease, *PD* progressive disease, *met*-*ADC* metastatic adenocarcinoma, *FISH* fluorescence in situ hybridization, *CCRT* concurrent chemoradiotherapy, *PTA* percutaneous transluminal angioplasty

Among 26 patients treated with crizotinib, we performed serial collection of blood samples in 12 patients. Four patients were initially positive and eight patients were negative for liquid biopsy. Dynamic change of ALK status in liquid biopsy was as follows: sustained positive (*n* = 1), positive conversion (*n* = 5), sustained negative (*n* = 3), negative conversion (*n* = 3). Most of the blood samplings were performed earlier from tissue diagnosis (median, 1.5 months, range 0.0–21.2) in patients available for serial monitoring than in those without serial collection (median, 14.5 months, range 0.0–120.0). Overall response to crizotinib of 12 patients was 50.0% (6/12) and disease control rate was 91.7% (11/12). Median PFS was 5.4 months (95% CI 5.3–5.6), and patients with positive results (sustained positive and positive conversion) showed a numerically shorter median PFS (5.4 months, 95% CI 1.9–8.8) that those with negative result (sustained negative and negative conversion, 10.5 months, 95% CI 0.0–21.4, p = 0.174). Representative cases are presented in Fig. 4.

Figure 4a details a case of a 52-year-old woman with stage IV ALK-positive NSCLC who had multiple metastases to the right pleura, both adrenal glands, and the brain. She showed continuous positivity in liquid biopsy during crizotinib treatment, but negative conversion was developed after debulking surgery for colon metastasis followed by alectinib treatment. In this case, the positivity of liquid biopsy was correlated in order with the clinical course.

Figure 4b details a case of a 67-year-old woman with lung-to-lung metastases. Her tumor was ALK-positive NSCLC and showed dramatic regression after crizotinib treatment. Liquid biopsy showed continuous negativity after the initial positive result only in platelets, and the results were correlated with findings of computed tomography scanning as a partial remission without recurrence.

Figure 4c details a case of a 78-year-old woman who was initially diagnosed with stage IIIB ALK-positive NSCLC. Liquid biopsy was negative after concurrent chemoradiotherapy (CCRT). However, upon completion of CCRT, right-sided hemiparesis was developed with multiple brain metastases. After receiving stereotactic brain radiotherapy (gamma-knife radiosurgery), she was treated with crizotinib, and negativity of liquid biopsy was maintained until 3 months before brain recurrence. After positive conversion, positive results of liquid biopsy were continued, along with recurrent brain metastasis; whereas, the intrathoracic lesion still had no evidence of recurrence.

## Discussion

In this study, we investigated the feasibility of blood-based liquid biopsy for the detection of *ALK* rearrangement and its predictive value for ALK inhibitor treatment. Liquid biopsy using plasma and platelets had favorable sensitivity compared with FISH assays for tumor tissues, and delayed blood sampling, since diagnosis showed higher positivity for liquid biopsy. In addition, platelets could predict the treatment outcome of ALK inhibitors more precisely than plasma.

Blood-based liquid biopsy is minimally invasive, easily repeatable method, and may predict acquisition of resistance by serial monitoring earlier than radiologic progression or the appearance of clinical symptoms. However, recent guidelines for liquid biopsy refer only to a limited subset of ALK-positive NSCLC (Merker et al. 2018; Rolfo et al. 2018). PCR-based methods are not recommended for routine use for *ALK* rearrangement detection from circulating tumor DNA (ctDNA). Platelet- or circulating tumor cell (CTC)-derived RNA may be useful; however, validation with a prospective cohort is necessary (Rolfo et al. 2018). This could be a problem owing to the source of liquid biopsy and the detection platform. The detection of CTCs is not routinely performed, and the technique has not been standardized. Circulating-free DNA requires extensive deep sequencing of genomic DNA for detection of the chromosomal break-point (Nilsson et al. 2016). Moreover, standardized methods for the isolation and analysis of extracellular vesicles are also needed (Sáenz-Cuesta et al. 2015). Digital-droplet PCR (ddPCR), BEAMing, and NGS have been reported to have promising sensitivity in the detection of *ALK* rearrangement, although there are still barriers for their use in daily practice.

Plasma and platelets have advantages in terms of easy isolation and smooth application to real practice, although they are not routinely isolated in clinics. In addition, RT-PCR can facilitate easy access, rapid readout, repeated examination, and satisfactory costs. In the present study, liquid biopsy using plasma and platelets analyzed by RT-PCR showed favorable performance in the detection of *ALK* rearrangement. In particular, platelets showed slightly higher sensitivity in detection and superior predictability of treatment outcomes than plasma. In addition, platelets showed better performance in the graph of Ct analysis than plasma. That is, platelets could better reflect the molecular status of tumor tissue than plasma, although there were no differences in total amount of extracted RNA between plasma and platelets. In several reports, platelets were found to contain the genetic components of primary tumors and metastatic lesions by uptake of tumor-derived RNA as a form of microvesicles, and it may be possible to provide more information on the tumor, suggesting the presence of tumor-educated platelets (Best et al. 2015; Joosse and Pantel 2015; Nilsson et al. 2016). However, the number of patients positive for liquid biopsy included in present study is small (*n* = 26), and those patients received crizotinib in different lines of treatment. Thus, a multivariate analysis in a large cohort of patients is needed to confirm a predictive power of ALK positivity in platelets for ALK inhibitor treatment.

However, the results of this study that patients positive for genetic alterations (*ALK* rearrangements) in blood component (platelets) had a better prognosis to targeted therapy (ALK TKI) are not typical findings compared with previous studies, especially for *EGFR* mutation. There may be two speculations for that result: one is that the phenomenon could be specific for ALK-positive lung cancer, and the other is that cases with FISH-positive in tissue and ALK-negative in platelets were false positives of tissue FISH assay. Thus, prospective collection of tissue specimen and further investigation with large numbers of cases would be in need.

In a previous study, plasma RNA showed lower sensitivity than platelets for the detection of *ALK* rearrangement by RT-PCR, and the authors speculated that rapid degradation of free-circulating RNA or lack of free-circulating exosomes containing *ALK* rearrangement may be the cause (Nilsson et al. 2016). In contrast, in the present study, plasma showed almost equivalent performance to platelets for detection, and the combination of positive results for plasma and platelets had synergistic effects on increased sensitivity. This may be attributed to the efforts for precise control of quality in pre-analytical parameters, such as appropriate tubes to collect blood, short delay for transfer to laboratory procedures (within 2 h from venous sampling), established centrifugation protocols, and storage of samples in a freezer. Thus, plasma and platelets may have applications in liquid biopsy for the detection of *ALK* rearrangement. Despite these advantages, however, several factors can influence the RNA profiles, including platelet counts, extent of cancer dissemination, amount of blood collection, systemic inflammation, and cardiovascular events (Joosse and Pantel 2015).

We found that the subgroup of delayed blood sampling since diagnosis showed higher positivity for liquid biopsy. This difference may be related to the observation that most blood sampling was performed after initiation of systemic treatment and that various treatment modalities were introduced before crizotinib treatment. Thus, although the small number of samples at the time of diagnosis could not be ignored, an assumption that the load of *ALK* rearrangement increased in patients who have not been treated effectively with ALK inhibitors may be reasonable. In fact, the result of liquid biopsy using platelets showed more negativity when the patients were treated with ALK TKI rather than chemotherapy. Therefore, liquid biopsy can facilitate the diagnosis of *ALK*-rearranged NSCLC as a supplement to tissue biopsy, and the detection rate and utility of liquid biopsy could be higher in the later period or at progression than in the initial period since diagnosis. However, to validate sensitivity of ALK liquid biopsy as a screening tool at diagnosis, analyzing only the samples collected before ALK TKI therapy may be warranted.

In the present study, serial monitoring using liquid biopsy showed that the analysis results were correlated with the therapeutic response to ALK inhibitors, as determined by imaging analysis, and a positive result was observed prior to true radiologic progression. This suggested that RNA released into the blood by free form (plasma) or loading in microvesicles (platelets) could function as a communicator between tumor cells and their microenvironment or distant metastasis. In addition, these findings supported that blood-based analysis could be used to monitor the ongoing alterations in tumors and predict disease progression, allowing for earlier adjustment of the treatment approach. Indeed, according to recent studies, liquid biopsy may have clinical value for the management of patients with ALK-positive NSCLC in the near future (Dagogo-Jack et al. 2018; Yoda and Lin 2018). This method could also be used to detect acquired resistance mutations from ctDNA in the setting of progression after first-line ALK TKI treatment, representing the clonal evolution of acquired mutations, and guiding the selection of subsequent ALK inhibitors. However, no studies have evaluated PCR-based platforms for resistance mutations in ALK-positive NSCLC. According to a recent guideline for liquid biopsy, an NGS panel using ctDNA is preferred for detection of *ALK* acquired resistance mutations when rebiopsy of the progression site is not feasible (Rolfo et al. 2018). Thus, prospective validation studies using RT-PCR may be warranted as a time-saving strategy for identification of mutation-specific inhibitory characteristics to facilitate the application of ALK inhibitors.

There were several limitations to this study. First, prospective sample collection was performed only for some samples. The number of samples at the time of diagnosis was not sufficient, and a prospective validation study needs to be performed to identify the role of ALK liquid biopsy in initial screening. Second, the total extracted amount of RNA from plasma and platelets was much lower than that from FFPE tissues. This could have been related to variations in tumor characteristics (i.e., shedding or non-shedding) as well as patient factors. Although direct application of the liquid source to a tissue-based kit showed favorable performance in this study, novel, more sensitive platforms for liquid-based PCR or high-end detection methods, such as ddPCR or NGS, are needed for screening and acquired mutation detection in ALK-positive NSCLC. Third, quantification of the results of RT-PCR was not performed, and such results may be crucial for predicting true progression during longitudinal monitoring. Finally, the results of genotyping for *EML4*-*ALK* fusion variants were not presented in this article. In recent studies, *ALK* variant status was found to affect the efficacy of ALK inhibitors, survival, and development of specific resistance mutations (e.g., variant 3 for G1202R mutation) (Lin et al. 2018; Woo et al. 2017; Yoshida et al. 2016). Indeed, we performed liquid-based genotyping for *ALK* variants using a co-developed tissue-based genotyping kit, but failed to derive meaningful data due to the high rates of invalid results (plasma, 46.8%; platelets, 53.4%; data not shown in tables). Further prospective validation studies with sensitive detection methods or other sources of liquid biopsy are necessary.

In conclusion, plasma and platelets are valuable and complementary sources for liquid biopsy in the detection of *ALK* rearrangements and showed favorable sensitivity, despite using a tissue-based RT-PCR kit. Liquid biopsy may have a supplementary role in the diagnosis of ALK-positive NSCLC, particularly during the later period after diagnosis, and platelets may be useful for predicting treatment outcomes of ALK inhibitors.
